# Attitudes toward smoking cessation according to smoking status among dentists in the Aichi Dental Association in Japan

**DOI:** 10.18332/tid/191290

**Published:** 2024-08-06

**Authors:** Yukie Oya-Watanabe, Koji Inagaki, Takahiro Nimi, Yohei Yamamoto, Toshiya Tanabe, Makoto Okai, Nobuhiro Segawa, Toshiyuki Watanabe, Noriyasu Uchibori, Tatsuro Koide, Junko Inukai, Hidemichi Yuasa, Akio Mitani, Toru Nagao, Makoto Fukui, Daisuke Hinode

**Affiliations:** 1 Department of Dental Hygiene, Aichi Gakuin University Junior College, Nagoya, Japan; 2 Department of Hygiene and Oral Health Science, Tokushima University Graduate School of Biomedical Sciences, Tokushima, Japan; 3 Department of Periodontology, School of Dentistry, Aichi Gakuin University, Nagoya, Japan; 4 The Aichi Dental Association, Nagoya, Japan; 5 Oral and Maxillofacial Surgery, Toyohashi Medical Center, Toyohashi, Japan; 6 Department of Maxillofacial Surgery, School of Dentistry, Aichi Gakuin University, Nagoya, Japan

**Keywords:** smoking cessation, smoking, dentists, heated tobacco product, graduate education

## Abstract

**INTRODUCTION:**

The prevalence of smoking, including heated tobacco products (HTPs), among Japanese dentists was reported to be 16.5%, significantly higher than that among Japanese physicians and United States dentists. However, large-scale studies on smoking cessation implementation based on dentists' smoking status and perceptions since the introduction of HTPs are lacking. Therefore, we aimed to investigate and assess dentists' attitudes toward smoking, including HTP use and smoking cessation, according to smoking status.

**METHODS:**

A self-administered questionnaire comprising six major items was mailed to 3883 dentists who were members of the Aichi Dental Association in August 2019. The primary outcome was smoking cessation status. The secondary outcome was the impact of smoking on intervention for smoking cessation. This study was reported using the Strengthening the Reporting of Observational Studies in Epidemiology guidelines.

**RESULTS:**

Among the 1317 (42%) dentists analyzed, men were more positive toward smoking than women. Current and former smokers were more positive about smoking than never smokers/users, regardless of the tobacco product type. Additionally, the current smoker group using conventional cigarettes was less likely to ask for their patients' smoking status than the never smoker group. Furthermore, the current smoker (OR=2.0; 95% CI: 1.3–3.1 vs never smoker) and HTP user (OR=1.9; 95% CI: 1.2–3.1 vs never user) groups were less likely to engage in smoking cessation than the never smoker/user groups, regardless of the tobacco product type.

**CONCLUSIONS:**

Since the smoking status of dentists affects the implementation of smoking cessation interventions, it is crucial to encourage them to quit using all tobacco products to promote smoking cessation interventions in dental practice. Additionally, providing proper smoking prevention education to dentists is an important task.

## INTRODUCTION

The prevalence of smoking, including heated tobacco products (HTPs), among dentists in Japan was reported to be 16.5%^1^, significantly higher than that among physicians (6.1%) in Japan in 2020^2^ and dentists (4.0%) in the United States in 2018^3^. A previous study reported that physicians who smoke are reluctant to quit smoking^4^. Consequently, smoking among dentists may hinder efforts to promote smoking cessation and have adverse effects on the health of their patients.

The World Dental Federation launched the tobacco cessation project in 2020, which also targets the use of HTPs. This initiative aims to engage oral healthcare professionals in smoking cessation efforts and provide resources that can be integrated into dental practices in collaboration with other relevant health professionals^5,6^. According to the guidelines, dental professionals play a crucial role in encouraging smokers to quit by ‘being a role model for their own health’, ‘providing guidance on smoking cessation’, ‘providing science-based information’, and ‘providing leadership’ as professionals. The United States has recognized the requirement for tobacco control in dentistry^7^ and implemented smoking cessation interventions by dental professionals^8^. However, insurance coverage for smoking cessation is limited to medical treatment in Japan despite smoking cessation being covered by health insurance since 2006. In the fields of medicine and dentistry, various guidelines have been proposed to promote tobacco control and smoking cessation among patients^9,10^. More than 60% of tobacco users visit a dentist or dental hygienist annually in developed countries; thus, oral healthcare providers have a wider reach among tobacco users and have great potential to persuade them to cease smoking^11^. Despite reports on the implementation of smoking cessation in the field of dentistry and its effects on periodontal therapies^12-14^, there have been no large-scale studies on the implementation of smoking cessation based on dentists’ smoking status and their perceptions of smoking cessation since the advent of HTPs. To promote effective smoking cessation in dentistry, it is important to understand the dentists’ attitudes toward smoking cessation, which may further contribute to improvements in the patient’s health. Therefore, in this study, we aimed to investigate the attitudes of Japanese dentists toward smoking cessation, particularly those in the Aichi Prefecture, where HTPs were first introduced in the country, in relation to their smoking status.

## METHODS

### Study design and participants

The survey was conducted from 20 August to 20 September 2019. Only completed questionnaires indicating consent to participate were included in this study. The research protocol adhered to the guidelines of the Declaration of Helsinki. In August 2019, a self-administered questionnaire on smoking cessation, adapted from parts of the questionnaire used among members of the Japanese Society of Periodontology^15^, was mailed to 3883 member dentists of the Aichi Dental Association. Of these, 3667 were male dentists.

### Sample-size calculation

The 2019 National Health and Nutrition Survey reported that the smoking rate among men was 27.1%, with approximately 30% of them being users of HTPs^16^. Therefore, the sample size of this study was determined to be 1153 using the statistical software EZR (Saitama Medical Center, Jichi Medical University, Saitama, Japan)^17^, assuming a smoking rate and confidence level of 25% and 95%, respectively.

### Primary outcome

The primary outcome of smoking cessation status among dentists was determined based on their responses to the question: ‘Do you provide smoking cessation guidance to patients who smoke?’. Implementation status was categorized as implementation for ‘yes’ responses and no implementation for ‘no, but I want to’ and ‘no, never’ responses.

### Secondary outcome

The secondary outcome was the impact of smoking on smoking cessation, measured as the degree of influence of smoking on intervention for smoking cessation. Smoking cessation implementation was examined according to smoking status.

### Definitions of cigarette smoking and/or HTP use

For cigarettes, individuals who had never smoked were classified as never smokers, those who had previously smoked but had completely quit were classified as former smokers, and those who smoked at least one cigarette per month were classified as current smokers. For HTPs, individuals who had never used HTPs were classified as never users, those who had previously used HTPs but had completely quit were classified as former users, and those who used HTPs at least once per month were classified as current users^1^.

Additionally, exclusive users of either HTPs or traditional cigarettes were defined as those who currently used only one of the two products. Dual users were defined as individuals who currently used both products^1^.

### Data collection

Data were collected through self-administered anonymous responses. Volunteers agreed to participate in the study and completed the questionnaire after receiving a written explanation about the study. Participants were informed that they could withdraw from the study at any time. All data were anonymized and kept strictly confidential.

### Statistical analysis

Data were analyzed using IBM SPSS Statistics for Windows, version 28 (IBM Corp., Armonk, NY, USA). The independent sample chi-squared test was used to compare the attitudes of the participants toward the smoking status of dental professionals and patients according to sex. After adjusting all variables for sex and age, a logistic regression analysis was conducted. The characteristics of the never smoker/user group were compared with those of the group that used only conventional cigarettes, the group that used only HTPs, and the dual-user group. Additionally, the impact was evaluated using odds ratios (OR) based on never smoker/user group. The goodness of fit of the final model was evaluated using the Hosmer-Lemeshow test, and OR with 95% confidence intervals (CIs) were calculated to assess the associations. The significance test was bilateral, and a p<0.05 was considered statistically significant. This study adhered to the Strengthening the Reporting of Observational Studies in Epidemiology guidelines for cross-sectional studies^18^.

## RESULTS

### Sample characteristics

Overall, 1617 dentists participated in the study (participation rate 41.6%, valid response rate 80.4%). Among them, the data from 1301 dentists who provided complete information regarding age, conventional cigarette smoking status, and attitudes toward the smoking status of healthcare professionals and patients in the questionnaire were analyzed (response rate, 80.4%; male dentists, 94.2%). Most participants were practicing dentists (94.2%) and aged > 50 years (20–49 years, 30.6%; ≥50 years, 69.4%). The smoking status of the participants was as follows: never smokers/users, 557; exclusive cigarette smokers, 103; exclusive HTP users, 66; and dual users, 46 (Table 1).

**Table 1:** Characteristics and smoking status of the participants

*Characteristics*	*Total (N=1301)*		*Men (N=1226)*		*Women (N=75)*	
	*n*	*%*	*n*	*%*	*n*	*%*
**Age** (years)						
20–29	5	0.4	5	0.4		
30–39	119	9.1	111	9.1	8	10.7
40–49	275	21.1	252	20.6	23	30.7
50–59	389	29.9	367	29.9	22	29.3
60–69	404	31.1	387	31.6	17	22.7
≥70	109	8.4	104	8.5	5	6.7
**Dental education level**						
Non-specialist	1028	80.7	963	80.3	65	86.7
Specialist	246	19.3	236	19.2	10	13.3
**Employment status**						
General practitioner	1,223	94.2	1169	94.5	67	89.3
Working as an employee	69	5.3	70	5.1	7	9.3
Leave of absence	6	0.5	6	0.4	1	1.3
**Cigarette smoking status**						
Never	558	42.9	495	40.4	63	84.0
Former	594	45.7	584	47.6	10	13.3
Current	149	11.5	147	12.0	2	2.7
**HTP using status**						
Never	842	64.7	773	63.1	69	92.0
Former	58	4.5	57	4.6	1	1.3
Current	112	8.6	112	9.1	0	0
**Status and pattern of smoking**						
Never smoker/user	557	42.8	494	40.3	63	84.0
Dual user	46	3.5	46	3.8	0	0.0
Exclusive cigarette smoker	103	7.0	101	8.2	2	2.7
Exclusive HTP user	66	2.8	66	5.4	0	0

HTP: heated tobacco product.

### Dentists’ attitude towards the smoking status of healthcare professionals and patients

Tables 2 and 3 present the results of the participants’ attitudes toward the smoking status of healthcare professionals and patients. Male participants (13.4%; 95% CI: 11.6–15.4) showed a more positive attitude toward smoking among healthcare professionals than female participants (5.2%; 95% CI: 1.6–13.0) (p<0.05) (Table 2).

**Table 2:** Dentists’ attitudes toward the smoking status of healthcare professionals and patients smoking and smoking cessation

*Attitudes*	*Total (N=1301)*		*Men (N=1226)*		*Women (N=75)*		*p*
	*n*	*% (95% CI)*	*n*	*% (95% CI)*	*n*	*% (95% CI)*	
**Smoking status of healthcare professionals**							
Yes (should not smoke)	905	69.4 (66.9–71.9)	843	68.6 (65.9–71.1)	62	83.1 (73.1–90.0)	0.022
No (personal freedom)	168	12.9 (11.2–14.8)	164	13.4 (11.6–15.4)	4	5.2 (1.6–13.0)	
Do not know	228	17.7 (15.7–19.8)	219	18.1 (16.0–20.3)	9	11.7 (6.1–21.0)	
**Smoking status of the patient**							
Yes (should not smoke)	706	54.1 (51.4–56.8)	661	53.8 (51.0–56.5)	45	59.2 (48.0–69.6)	0.206
No (personal freedom)	558	43.1 (40.4–45.8)	532	43.6 (40.8–46.3)	26	35.5 (25.7–46.8)	
Do not know	37	2.8 (2.0–3.9)	33	2.7 (1.9–3.7)	4	5.3 (6.2–24.1)	
**Smoking status of patients with periodontal disease**							
Yes (should not smoke)	868	66.7 (64.1–69.2)	813	66.3 (63.6–68.9)	55	74.0 (63.2–82.6)	0.296
No (personal freedom)	407	31.3 (28.8–33.8)	389	31.8 (29.2–34.4)	18	23.4 (15.2–34.0)	
Do not know	26	2.0 (1.3–2.9)	24	1.9 (1.3–2.9)	2	2.6 (0.2–9.5)	
**Do you ask all patients about their smoking status and history?**							
**Yes**							
All patients	351	27.0 (24.6–29.4)	319	26.0 (23.6–28.5)	32	42.7 (31.1–53.2)	0.003
More than half of patients	312	24.0 (21.7–26.4)	293	23.9 (21.6–26.4)	19	25.3 (15.5–35.4)	
**No**							
A small percentage of patients	336	25.8 (23.5–28.2)	319	26.0 (23.7–28.5)	17	22.7 (13.9–32.8)	
Completely	301	23.2 (20.8–25.5)	294	24.0 (21.6–26.3)	7	9.3 (3.0–16.7)	
**Do you ask all patients with periodontal disease about their smoking status and history?**							
**Yes**							
All patients	420	32.3 (29.8–34.8)	380	31.0 (28.6–33.5)	40	53.3 (41.2–64.3)	0.001
More than half of patients	327	25.2 (22.9–27.5)	305	24.9 (22.6–27.3)	22	29.3 (19.2–39.4)	
**No**							
A small percentage of patients	323	24.8 (22.7–27.2)	313	25.6 (23.0–28.2)	10	13.3 (6.2–21.6)	
Completely	230	17.7 (15.5–19.8)	227	18.5 (16.2–20.7)	3	4.0 (0.0–9.2)	
Not answered	1		1		0		
**Do you provide smoking cessation guidance to patients who smoke?**							
**Yes**							
Performed on all patients	70	5.4 (4.2–6.6)	65	5.3 (4.0–6.7)	5	6.8 (1.6–12.7)	0.018
Performed on patients who need it	540	41.6 (38.8–44.3)	498	40.7 (37.8–43.4)	42	56.8 (45.5–67.5)	
No, but I want to do	395	30.4 (27.7–33.0)	376	30.7 (28.1–33.3)	19	25.7 (15.4–36.4)	
No, never	294	22.6 (20.3–24.9)	286	23.3 (20.8–25.9)	8	10.8 (4.3–17.8)	
Not answered	2		1		1		

Chi-squared tests were used to evaluate the sex differences.

**Table 3:** Multiple logistic regression examining the dentists’ attitudes toward the smoking status of healthcare professionals and patients according to the smoking/using status

*Variable*	*Dentists’ attitudes toward*					
	*Smoking status of healthcare professionals (Ref. yes)*		*Smoking status of patients (Ref. yes)*		*Smoking status of patients with periodontal disease (Ref. yes)*	
	*n*	*AOR (95% Cl)*	*n*	*AOR (95% Cl)*	*n*	*AOR (95% Cl)*
**Cigarette smoking status**						
Never ®	41	1	202	1	140	1
Former	78	2.0 (1.3–3.0) ***	250	1.2 (1.0–1.6)	181	1.3 (1.0–1.7)
Current	49	9.0 (5.4–14.9) ***	106	4.2 (2.8–6.4) ***	86	4.0 (2.7–5.9) ***
**HTP using status**						
Never ®	108	1	437	1	310	1
Former	17	4.8 (2.5–9.1) ***	39	3.3 (1.8–5.8) ***	32	3.3 (1.9–5.6) ***
Current	43	10.1 (6.1–16.7) ***	82	4.9 (3.1–7.8) ***	65	4.1 (2.7–6.1) ***
**Smoking/using status**						
Never smoker/user ®	41	1	202	1	141	1
Dual user	19	16.5 (7.5–36.6) ***	33	4.5 (2.3–8.9) ***	29	5.1 (2.7–9.7) ***
Exclusive cigarette smoker	30	7.2 (4.1–12.6) ***	73	4.1 (2.6–6.6) ***	57	3.6 (2.3–5.6) ***
Exclusive HTP user	24	12.5 (6.3–24.7) ***	49	6.2 (3.3–11.7) ***	36	4.0 (2.3–7.0) ***

The adjusted odds ratio (AOR) represents the results of the multivariable logistic regression analysis adjusted for sex and age groups. HTP: heated tobacco product. ® Reference categories.

*** p<0.001.

Furthermore, current smokers (OR=9.0; 95% CI: 5.4–14.9 vs never smoker)/users (OR=10.1; 95% CI: 6.1–16.7 vs never user) and former smokers (OR=2.0; 95% CI: 1.3–3.0 vs never smoker)/users (OR=4.8; 95% CI: 2.5–9.1 vs never users) were more positive towards healthcare professionals’ smoking than those who have never smoked or used tobacco products (p<0.05) (Table 3). Moreover, regarding patients’ smoking, current smokers (OR=4.2; 95% CI: 2.8–6.4 vs never smokers)/users (OR=4.9; 95% CI: 3.1–7.8 vs never users) were more positive than those who have never smoked or used tobacco products (p<0.05) (Table 3). Based on smoking/using status, dual users (OR=16.5; 95% CI: 7.5–36.6 vs never smoker/users) were most positive towards healthcare professionals’ smoking, while exclusive HTP users (OR=6.2; 95% CI: 3.3–11.7 vs never smoker/users) were most positive towards patients’ smoking (p<0.05) (Table 3).

### Status of smoking cessation

**Figure 1:**
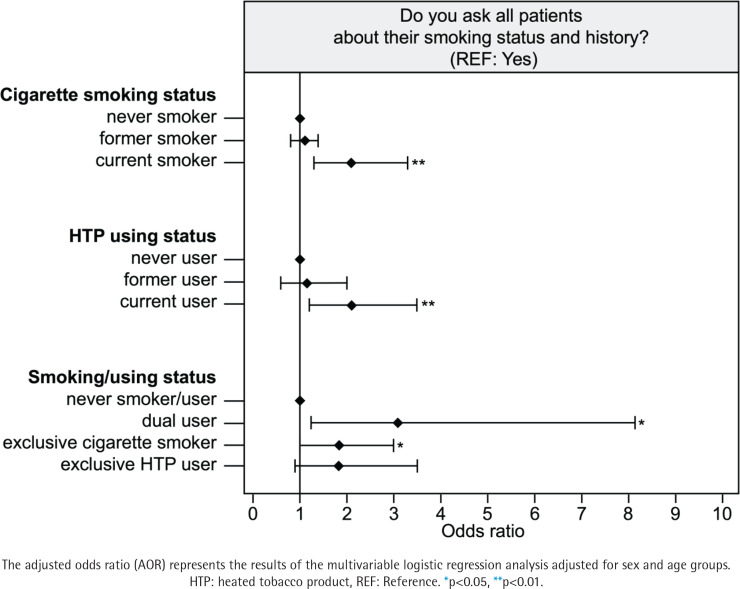
Assessment of patients’ smoking status based on dentists’ smoking status

**Figure 2:**
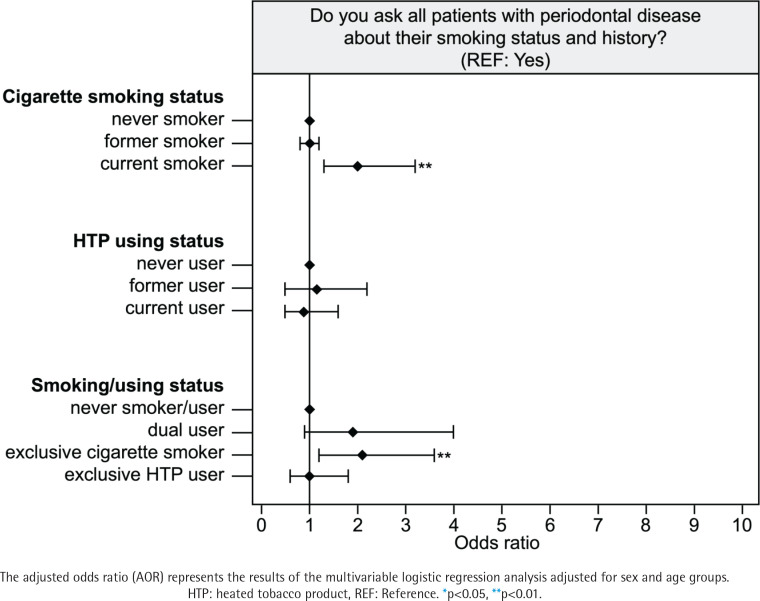
Assessment of the smoking status of patients with periodontal disease based on dentists’ smoking status

**Figure 3:**
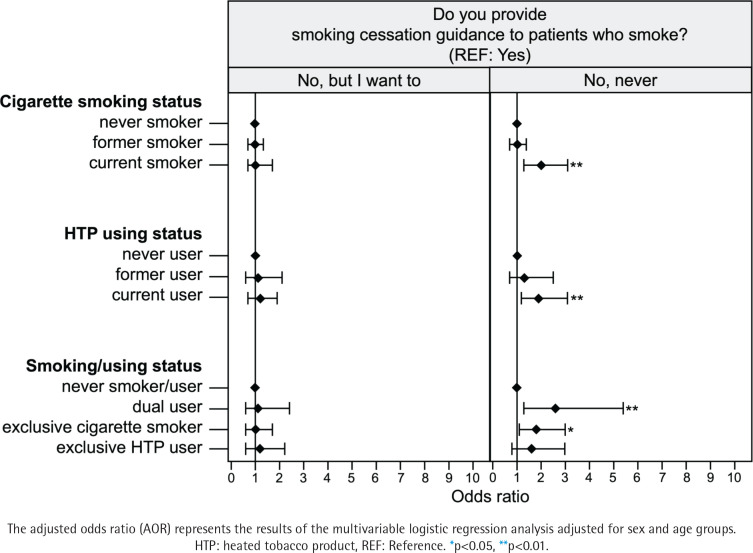
Dentists’ willingness to provide smoking cessation support by smoking status

Male participants were more reluctant to quit smoking than female participants (p<0.05) (Table 2). Participants in the current smoker group who were conventional cigarette smokers (OR=2.1; 95% CI: 1.3–3.3 vs never smoker) were less aware of the smoking status of their patients than those in the never smoker group (p<0.05) (Table 4). Furthermore, participants in the current smoker (OR=2.0; 95% CI: 1.3–3.1 vs never smoker) and user groups (OR=1.9; 95% CI: 1.2–3.1 vs never user) were less likely to engage in smoking cessation than those in the never smoker and user groups, regardless of the type of tobacco product used (p<0.05) (Table 4). More details are given in Figures 1–3.

**Table 4:** Multiple logistic regression examining the status of smoking cessation by smoking/using status

*Variable*	*Do you ask all patients about their smoking status and history? (Ref. yes)*		*Do you ask all patients with periodontal disease about their smoking status and history? (Ref. yes)*		*Do you provide smoking cessation guidance to patients who smoke? (Ref. yes)*			
					*No, but I want to*		*No, never*	
	*n*	*AOR (95% Cl)*	*n*	*AOR (95% Cl)*	*n*	*AOR (95% Cl)*	*n*	*AOR (95% Cl)*
**Cigarette smoking status**								
Never ®	386	1	360	1	169	1	113	1
Former	428	1.1 (0.8–1.4)	390	1.0 (0.8–1.2)	182	1.0 (0.7–1.3)	127	1.0 (0.7–1.4)
Current	122	2.1 (1.3–3.3) **	117	2.0 (1.3–3.2) **	78	1.0 (0.7–1.7)	51	2.0 (1.3–3.1) **
**HTP using status**								
Never ®	800	1	749	1	340	1	239	1
Former	43	1.1 (0.6–2.0)	38	1.2 (0.5–2.2)	18	1.1 (0.6–2.1)	15	1.3 (0.7–2.5)
Current	93	2.1 (1.2–3.5) **	80	0.9 (0.5–1.6)	32	1.2 (0.7–1.9)	37	1.9 (1.2–3.1) **
**Smoking/using status**								
Never smoker/user ®	385	1	359	1	169	1	112	1
Dual user	39	3.1 (1.2–8.1) *	35	1.9 (0.9–4.0)	11	1.1 (0.5–2.4)	18	2.6 (1.3–5.4) **
Exclusive cigarette smoker	83	1.8 (1.0–3.0) *	82	2.1 (1.2–3.6) **	28	1.0 (0.6–1.7)	33	1.8 (1.1–3.0) *
Exclusive HTP user	54	1.8 (0.9–3.5)	45	1.0 (0.6–1.8)	21	1.2 (0.6–2.2)	19	1.6 (0.8–3.0)

The adjusted odds ratio (AOR) represents the results of the multivariable logistic regression analysis adjusted for sex and age groups. HTP: heated tobacco product. ® Reference categories.

* p<0.05,

** p<0.01.

## DISCUSSION

### Dentists’ attitudes towards the use of HTPs among healthcare professionals and patients

According to the 2020 Japan Medical Association (JMA) survey, awareness of smoking has increased steadily over the past 20 years among physicians and patients, with 80% of JMA members expressing a negative attitude toward smoking among physicians and 60% expressing a negative attitude toward smoking in general^19^. A 2009 survey of periodontists reported similar trends, with 80% of dentists and approximately 60% of patients expressing disapproval of smoking. Notably, current smokers were more tolerant of both concepts^20^.

In this study, although female participants comprised a smaller percentage of the total, they showed a more negative attitude toward smoking among healthcare professionals than male participants. Additionally, former and current smokers were more positive about smoking among healthcare professionals and patients than never smokers, consistent with the findings of previous studies^21,22^. Furthermore, similar results were obtained regarding the use of HTPs, showing for the first time that current smoking status is a factor influencing the attitude toward smoking, regardless of the smoking/HTP use status, type of tobacco product, or dual use. Despite the greater awareness of its risks in HTP users than in never smokers^1^, HTP users among healthcare professionals and patients were more positive towards smoking. Such results have not been found in previous studies. The World Health Organization states that dental professionals should be health role models themselves and provide evidence-based information to promote smoking cessation^5^. The results of this study suggest that the quality of dental care services is affected by the smoking status of the dentist, as dentists who are smokers may lose the opportunity to promote smoking cessation among patients. Particularly, patients who are smokers are less aware than never smokers that smoking exacerbates periodontal disease^23,24^; thus, oral health instructions that incorporate information on smoking cessation should be actively implemented. Therefore, the smoking status of dentists who provide smoking cessation support was considered an important factor in promoting smoking cessation.

### Smoking cessation interventions based on smoking status

The smoking status of physicians has been reported to influence the quality of motivation and content of smoking cessation interventions for patients who smoke^22,25^. Additionally, smoking cessation interventions were less frequently provided to patients who used e-cigarettes than those who smoked cigarettes^26^. In this study, conventional cigarette smokers and HTP users were approximately two times as likely as never smokers/users and 2.6 times more likely than dual smokers to not provide smoking cessation intervention. Thus, in addition to showing trends similar to those in previous studies, this study also suggested that smoking, including HTP use, is a factor that hinders smoking cessation guidance.

Since the formulation of the 1964 statement on interventions for tobacco use, the American Dental Association has been educating its members on implementing such interventions^27^. Additionally, the Office of Disease Prevention and Health Promotion has set the goal to increase the proportion of healthy adults receiving advice on smoking cessation from healthcare providers by 2030^28^ and is implementing an ongoing national smoking cessation strategy. In Japan, smoking cessation was first added to the model core curriculum for dental education in 2010 ^29^. However, since approximately 90% of the participants in this study were aged ≥40 years, the results reflected the experience of participants who had no opportunity to learn about smoking cessation as a part of their dental education. Furthermore, while smoking cessation has been covered by insurance in Japan since 2006, the coverage is limited to the medical field, and smoking cessation is excluded from dental insurance. This lack of coverage could have contributed to dentists’ reluctance to proactively provide smoking cessation advice.

In Japan, support for smoking cessation has been integrated into dental education for over a decade, although it is still at an earlier stage compared to that in the United States. Therefore, there is a need to enhance undergraduate and postgraduate education on smoking and smoking cessation, including the use of HTPs, to increase the implementation rate of smoking cessation support and provide better dental care to patients.

### Limitations

This study has some limitations. First, the status and challenges associated with smoking cessation were based on the responses obtained from the participants in this study, with a participation rate of only 41.6%. The situation in dental clinics that did not respond could not be explored; therefore, the actual implementation rate of smoking cessation may be lower than that reported in this study. Regardless, this study is significant as it highlighted the status of smoking cessation efforts in dentistry after the spread of the use of HTPs and yielded findings that are valuable to the implementation of tobacco control measures, including smoking cessation education for oral healthcare providers. Additionally, considering that women represent only 5.8% of the participation rate, future research should target dental hygienists, who have a higher proportion of female employees, to investigate their perceptions regarding smoking cessation among dental healthcare providers. Second, the questionnaire used in this study did not include items regarding income or education level beyond the required years of dental education. Therefore, we did not collect information on socioeconomic status and education level beyond dental education. Consequently, potential confounding factors such as socioeconomic status and education level were not accounted for in the analysis of this study. Future research should consider including more detailed confounding factors for a comprehensive analysis. Third, as the questionnaire did not include specifics regarding smoking cessation interventions, it was impossible to evaluate the validity of the content and quality provided by the dentists who indicated the provision of the respective interventions. Future studies should be conducted to clarify the actual smoking cessation interventions provided by dentists and the effect of these interventions on the incidence of patients’ smoking cessation.

## CONCLUSIONS

Since the smoking status of dentists affects the implementation of smoking cessation interventions, it is crucial to encourage them to quit using all tobacco products to promote smoking cessation interventions in dental practice. Additionally, given that HTPs have been available in Japan for the past decade, it is a crucial task to provide appropriate smoking prevention education, including information on HTPs, as part of postgraduate education for dentists.

## Data Availability

The data presented in this study are available upon reasonable request from the corresponding authors.
